# Computational Tools for the Secondary Analysis of Metabolomics Experiments

**DOI:** 10.5936/csbj.201301003

**Published:** 2013-02-06

**Authors:** Sean C. Booth, Aalim M. Weljie, Raymond J. Turner

**Affiliations:** a Department of Biological Sciences, University of Calgary, Calgary, AB. 2500 University Dr. NW, Calgary, Alberta, T2N 1N4, Canada; b Department of Pharmacology, University of Pennsylvania, Philadelphia, United States

**Keywords:** Metabolomics, systems biology, functional genomics, computational analysis, software tools, metabolite enrichment

## Abstract

Metabolomics experiments have become commonplace in a wide variety of disciplines. By identifying and quantifying metabolites researchers can achieve a systems level understanding of metabolism. These studies produce vast swaths of data which are often only lightly interpreted due to the overwhelmingly large amount of variables that are measured. Recently, a number of computational tools have been developed which enable much deeper analysis of metabolomics data. These data have been difficult to interpret as understanding the connections between dozens of altered metabolites has often relied on the biochemical knowledge of researchers and their speculations. Modern biochemical databases provide information about the interconnectivity of metabolism which can be automatically polled using metabolomics secondary analysis tools. Starting with lists of altered metabolites, there are two main types of analysis: enrichment analysis computes which metabolic pathways have been significantly altered whereas metabolite mapping contextualizes the abundances and significances of measured metabolites into network visualizations. Many different tools have been developed for one or both of these applications. In this review the functionality and use of these software is discussed. Together these novel secondary analysis tools will enable metabolomics researchers to plumb the depths of their data and produce farther reaching biological conclusions than ever before.

## Introduction

Over the past decade metabolomics has emerged as a powerful tool used in a variety of quite diverse fields for hypothesis development, to elaborate unknown gene functions, biomarker discovery and to complement proteomic and transcriptomic experiments. While considerable progress has been made, the datasets obtained from metabolomics experiments still remain extremely large and dense and thus subsequently a challenge to interpret and derive biological meaning. This challenge lies in the difficulty of understanding how dozens of chemically diverse compounds, a small subset of the hundreds to thousands of metabolites present within cells, are functionally related to each other and the perturbed condition of the experiment. While it is possible, and common, for experimenters to intuitively interpret these results using their knowledge of metabolism and the tested conditions, or manually map them onto known metabolic pathways, computational analysis allows for more comprehensive interpretation. As metabolomics remains a developing field, bioinformatic tools designed to perform this task continue to be developed and released by various groups using diverse algorithms. While many databases, tools and projects such as the human metabolome database [1] have focused on creating tools specifically for interpreting human metabolomics experiments, the options for more diverse organism metabolomics are somewhat limited. This review seeks to introduce the problems faced when interpreting metabolomics results and describe the most current approaches to solving these problems in various model and experimental systems without a human centric bias.

## Background

The central dogma of molecular biology delineated the basic transfer of biological information as moving from DNA to RNA to protein [2]. While many proteins interact with each other and the nucleic acids, the real metabolic function of the cell relies on the enzymatic interconversion of the various small, low molecular weight compounds, termed metabolites[3]. These metabolites represent the actual functional phenotype of the cell that when systematically identified and quantified, the process of metabolomics, will show an accurate snapshot of the cell’s physiological state [4]. A relative newcomer to the ‘omics’ field compared to proteomics and transcriptomics, the technologies and techniques behind metabolomics have been evolving rapidly to even the point where commercial kits are available for common clinical samples [5]. While still a developing field, excellent reviews of topics in designing a metabolomics experiment from sample selection and preparation [6, 7], analytical techniques [4, 8] to data processing [9, 10] and statistics [11] are available. The frequent final product of the metabolomics pipeline is the generation of a list of metabolites who’s concentrations have been (significantly) altered which must be interpreted in order to derive biological meaning. While tools designed for this function exist, the development of many of these tools have been driven by the application of metabolomics to human pathologies such as kidney [12], heart [13], and neurological [14] disease and especially cancer [15, 16] leaving more broadly applicable tools lagging somewhat behind. Additionally there is no widely accepted standard for the computational interpretation of metabolite data whereas the interpretation of protein and transcript expression datasets is much more mature [17]. To fill these voids a number of tools have recently been developed with fresh ideas, providing new releases constantly, as this field emerges out of its adolescence. A challenge though is that as of yet, none have emerged as a standardized approach. Here, current solutions for metabolomic data interpretation will be described with reference to studies that have taken advantage of these new methods will be presented. Throughout, tools with a focus on those which can be broadly applied to any organism will be highlighted.

*Preprocessing*: Computational procedure where raw data (GC/LC-MS, NMR spectra) are converted into a useable form. Removes bias and makes samples comparable.

*Targeted metabolomics*: Directed measurement of a group of metabolites suspected to be relevant in a particular system.

*Untargeted metabolomics*: Quantification of as many metabolites as possible within the bounds of an instrument.

*Secondary Analysis*: Data interpretation procedure where a finalized dataset is subject to higher level analysis using information obtained from biochemical databases.

*Metabolic Pathway*: A series of enzyme-catalyzed biochemical reactions that bring a number of metabolites together under the umbrella of one particular biological function.

*‘Omics*: General term for the high through-put technologies that identify and quantify large groups of targets at once including transcriptomics (mRNA), proteomics (proteins) and metabolomics (metabolites).

*Unannotated compound*: A metabolite to which no biological function has been ascribed either in life in general or in the specific organism in question.

*Unknown compound*: A compound that produces a unique chromatographic peak and mass spectrum, but who’s structure and name are unknown.

Metabolomics requires many steps and choices before getting to the point of data interpretation which will affect how this process is undertaken. The main decisions are analytical platform (likely GC/LC-MS or NMR as they are the most common), each with their own advantages and disadvantages though the choice will more likely be dictated by instrument availability and analytical method (chemometric or quantitative) determined by the scope of the experiment. GC-MS is an extremely common metabolomics platform, resulting in a high frequency of tools which allow for the direct input of GC-MS spectra. The popularity of GC-MS is due to its relatively high sensitivity, broad range of detectable metabolites, existence of well-established identification libraries and ease of automation [18]. Even with its popularity, separation-coupled MS data requires much processing and careful handling to ensure the information it contains is not artifactual [19]. While scientists have been quantifying metabolite levels for over 50 years through targeted analysis, the focus here will be on *untargeted* metabolomics as the problem of interpretation mainly needs to be dealt with for this kind of experiment. Untargeted metabolomics aims to identify and quantify as many of the metabolites in a sample as possible then determine which are important, rather than focusing on identifying and quantifying a specific set of metabolites which are expected to be important (the targeted approach) [20]. When this approach is undertaken with one of the three most common instruments (GC/LC-MS or NMR) metabolites are identified by using pure reference spectra (plus chromatographic information if applicable) which also allow for quantification [10].These techniques, and others were reviewed by Zhang *et al*. [8].

Advances in instrumentation and technical treatment of samples as well as data preprocessing and development of improved databases have been arriving rapidly in metabolomics leading to ever increasing numbers of metabolites identified and accuracy of their quantification[8]. With these improvements, one would expect the results of metabolomics to have a profound effect on the questions they’re being applied to. Indeed metabolomics approaches have shown many successes in identifying potential therapeutic targets and also assigning function to unknown genes/proteins[20], thus effectively connecting to the field of functional genomics. Phenotype characterization studies however, such as in environmental metabolomics, often tend to be limited to speculating cause/effect relationships based on prior knowledge[21]. Many studies results‘ are discussed in terms of ‘suggestions’, ‘correlations’, or the individual metabolites changing are not even discussed, just the fact that discerning metabolic patterns are identifiable [22].This process of comparing metabolic profiles and only looking for differences is more exaggerated when metabolomics is used for biomarker discovery. This process of identifying specific metabolites that are altered in a disease state, as well as general metabolic differences is common in metabolomics studies of human pathologies [12, 15, 23]. Putative biomarkers are often then confirmed using a second dataset and/or by confirmatory experiments examining the metabolite in cell cultures[12]. While these methods have obvious and well-realized implications in the clinical field, current metabolomics interpretations, especially outside of human medicine, are generally over-reliant on additional research for explanations as well as providing underwhelming conclusions for data that purports to represent the basal level of functionality within the cell culture, tissue or organism. Fortunately, tools designed to better mine and interpret metabolomics data have been under rapid development recently. Indeed, this step has been called a ‘bottleneck’ in the metabolomics pipeline [24, 25, 26].

## Key Issues

It is in the final steps of interpretation where the most potential remains to improving the quality of information obtained from metabolomics [27, 28, 29, 30]. By this point though prior steps have created several problems which must be overcome when interpreting metabolomics data: 1) All of the metabolites within a system cannot be identified with any one analytical method due to chemical heterogeneity, which will cause downstream issues as all metabolites in a pathway have not been quantified; 2) not all metabolites have been identified and characterized and so do not exist in the standards libraries, leading to large number of unannotated and/or unknown metabolites of interest; 3) organism specific metabolic databases/networks only exist for the highest use model organisms making contextual interpretations difficult for many researchers; 4) interpreting the huge datasets of metabolite concentrations under various conditions with biological context is an inherently complex problem requiring extremely in depth knowledge of metabolism. There is also one final problem, the issue of determining which metabolites are actually important in the experimental system in question. While there is no standardized method for this, there are many statistical tests and tools available to researchers to pick out statistically significant metabolites from noise [11]. The remaining issues have fortunately already been and will continue to be addressed to varying degrees as advances in technologies and method developments rapidly evolve. The first three problems will generally be solved/alleviated over time as advances in instrumentation and their combined use as well as the continued curation and community development of databases allows for more metabolites to be identified in a more contextual fashion. The final problem, which is the main topic of this review, will only be solved as our understanding of systems biology evolves and tools to tap this knowledge keep up. The current generation of platforms, which are at the cutting edge of the field have generally been built upon the foundations laid by the large biochemical databases.

## Bioinformatic Basis

With the advent of the genomic age, the amount of biochemical knowledge has exploded in the last two decades which has necessitated its storage in large databases. A variety of top-down (gene to protein to metabolite) and bottom-up (chemical entity to biological function) approaches have been taken resulting in a rich expanse of metabolic knowledge bases available to query. These databases provide the contextual biochemical basis for metabolomics data interpretation. By supplying information about metabolites, such as defining which enzymatic reactions consume or produce them, and which pathways they’re involved in, researchers can use them to interpret their experiments to higher levels. An excellent review of these (top-down) types of databases is available in [31], while a more expanded review of databases is available in [32] and more recently in [33]. Also the Metabolomics Society website provides an excellent resource (www.metabolomicssociety.org/database). Additionally, and more specific to the development of metabolomics, mass spectral databases like the Golm Metabolome Database (GMD), which link mass spectrum and chromatographic retention time to specific compounds have been developed for use in the identification stages of metabolomics [34, 35]. Some tools designed for higher level metabolomic analysis can take GC-MS spectra as input and so have integrated select databases into their platform. The human metabolome database (HMDB)[1] warrants mention here as while it is highly specific, it contains integrated information from spectra (multiple NMR, GC-MS) to clinical relevance. As a result it has been integrated into several platforms. By far the major database that has been integrated into metabolomics interpretation platforms is the Kyoto Encyclopedia of Genes and genomes (KEGG), which is divided into several sub-databases with LIGAND, REACTION PAIR and PATHWAY being the most relevant to metabolomics [36]. These databases have been undergoing continuous updating and annotation for close to 20 years and so contain a great deal of valuable information. KEGG and MetaCyc are currently the largest (most number of organisms) and most in depth comprehensive (i.e. contains linked information from metabolite to gene) databases available, and so have been frequently integrated into interpretation platforms. The most commonly integrated databases have been summarized in Table 1.This leaves other databases (further reviewed in [31]), such as Reactome [37] (human),KNApSAcK [38] (plants), Model SEED [39] (diverse), and BiG [40] (6 model organisms), somewhat overshadowed, though they do have their own tools for use in metabolomic analysis, and can be more useful than the large databases if a specific organism is desired. The KEGG and MetaCyc databases each contain a generalized ‘conserved’ set of pathways based on metabolic pathways that are more or less the same throughout life in general. For KEGG, organism specific annotations are available to query while for MetaCyc, individual ‘Cyc’ databases have been generated for a number of organisms, some just computationally, others extensively manually curated such as AraCyc for *Arabidopsis* [41]. A more recent development are the cheminformatic databases like PubChem [42] and ChEBI [43], which provide a chemically ontological approach to cataloguing the ill-defined category of ‘small molecules’ active in biological systems. These types of databases can provide additional non-biology specific information as well alternative formatting options for datasets. Finally it is important to note that the few databases discussed here are by no-means exhaustive and that these databases are cross-referenced and linked to each other as well as against more widely known databases such as the well-known Chemical Abstract Service (CAS) [44] among many others.

**Table 1:** Selected Biochemical Databases

	KEGG^36^	MetaCyc [45]	PubChem [42]	ChEBI^43^	GMD^34^	HMDB^1^
Link	*www.genome.jp/kegg/*	*www.genome.jp/kegg/*	*pubchem.ncbi.nlm.nih.gov/*	*www.ebi.ac.uk/chebi/*	*gmd.mpimp-golm.mpg.de/*	*www.hmdb.ca/*
Type	Comprehensive	Comprehensive	Chemical	Chemical	Mass Spectral	Mass Spectral Comprehensive
Database Features	Genomes, genes, proteins, metabolites, drugs, diseases, pathways, visualizations	Genes, proteins, metabolites, pathways, interactive visualizations	Compound	Compound	Metabolites	Metabolites
Specificity	Generalized annotations, 2260 organism semi-specific annotations	Generalized annotations, 1939 organism specific annotations	Broad	Broad	Broad, plant heavy	Human

## Metabolomics Secondary Analysis: Enrichment Analysis and Metabolite Mapping

Biochemical databases provide an excellent backdrop of information for metabolomic analysis tools to query. Like many techniques in metabolomics, the algorithms for using these databases for interpretation evolved from methods developed for transcriptomic and proteomic analysis, such as Gene Set Enrichment Analysis (GSEA) [46]. This landmark technique has been the clear influence for several recent metabolomic tools, namely PAPi [24], MBRole [27], MSEA [30, 47] (as implemented by two different groups) and MPEA [29]. While each tool is unique in its algorithm, the general idea of enrichment analysis is used by all. Enrichment analysis depends on meta-data being associated with metabolites as biochemical entities. As such they can be annotated with various classifiers such as chemical family or which metabolic pathways it is involved in. Enrichment analysis can then take a list of metabolites, and with some tools their relative abundances (including positive/negative changes), and calculate based on some metric whether any particular pathway(s) (or some other classifier such as chemical family) is (statistically) more represented than any other, based on all possibilities. The assumption is then that this particular pathway is being more perturbed by the experimental condition than others, hence the observed significance and alterations to concentrations in the input metabolites. This method of secondary analysis has evolved alongside the complementary technique of metabolite mapping of which available non-specific network visualization tools have been reviewed in [48, 49, 50].These generic network tools allow for integration of multiple ‘omics datasets, as well as more user controlled flexibility. Metabolomics specific network mapping tools also exist, some of which are components of databases such as the KEGG pathway databases (KEGG Atlas) and MetaCyc’s Pathway Tools [51, 52]. Other explicitly designed tools are also available, some of which have been summarized in Table 2. CytoScape[53] is a highly used/integrated stand-alone networking program for ‘omics datasets which even has plugins like MetScape[54] designed for viewing human metabolic data. The principal idea behind pathway mapping is the contextual visualization of metabolomics data. On these networks, nodes represent metabolites and edges (connecting lines) represent enzymatic conversions. By highlighting the significantly changed metabolites (with or without magnitudes) on organism specific (if available) or life-general metabolic pathways a researcher is provided with an interpretable visualized representation of their data. Biological inferences can then be made by manually inspecting these figures, while some platforms provide network topology analysis tools. The complex subject of computational representation and analysis of metabolic networks has been reviewed in [55]. Between visualization and enrichment analysis secondary analysis is becoming an important step in biological interpretation of metabolomics experiments.

**Table 2:** Selected Platforms for Metabolomics Analysis and Interpretation

Name	Link	Access	Input	Databases Used	Functions	Comments
MetExplore [28]	http://metexplore.toulouse.inra.fr	Web-based	Compound IDs, Mass IDs	Generally BioCyc related	Compound mapping, graph analysis of metabolism maps.	Choice of organism database, filtering options, multiple graph analysis tools, Cytoscape integration.
PAPi [24]	http://www.4shared.com/file/0v5zSobM/PAPi_10.html	R Package	KEGG Compound IDs	KEGG	Compares activity of metabolic pathways between sample types.	Non organism specific, more difficult/powerful command line R interface. Usable with spent media results.
MBRole [27]	http://csbg.cnb.csic.es/mbrole/	Web-based	Compound IDs	KEGG, HMDB, PubChem, ChEBI, SMILES	Enrichment analysis of metabolites’ annotations.	Background set from known organisms or custom set. Metabolite ID converter.
MetaboAnalyst[57]	http://www.metaboanalyst.ca/MetaboAnalyst/	Web-based	Raw Spectra (GC and LC MS), peak lists and spectral bins (MS and NMR)	Custom, KEGG, HMDB	Full processing, Statistical Analysis	Comprehensive metabolomics analysis platform with easy interface, tutorials, help. Human focused though some model organisms or custom metabolite set option.
MetaboAnalyst(MSEA) [58]	http://www.metaboanalyst.ca/MetaboAnalyst/	Web-based.	Compound IDs and abundances	Custom, KEGG, HMDB	Enrichment Analysis	Comprehensive metabolomics analysis platform with easy interface, tutorials, help. Human focused though some model organisms or custom metabolite set option.
MetaboAnalyst (MetPa [59])	http://www.metaboanalyst.ca/MetaboAnalyst/	Web-based	Compound IDs and abundances	KEGG	Pathway Analysis	Select model organisms. Network topology analysis. Intuitive network visualization.
MPEA [29]	http://ekhidna.biocenter.helsinki.fi/poxo/mpea/	Web-based	Compound IDs, GC-MS Spectrum as ranked list	KEGG, GMD, SMPDB	Pathway enrichment analysis.	Optional background set. Limited to top-down/bottom-up analysis.
MeltDB (MSEA) [30, 60]	http://www.cebitec.uni-bielefeld.de/groups/brf/software/meltdb_info/	Web-based, login required	Raw GC/LC-MS spectra, processed spectra, compound IDs and abundances	GMD, KEGG, ChEBI, CAS	Comprehensive preprocessing, statistical analysis and metabolite mapping, enrichment analysis.	Integrated comprehensive online system, accessible by multiple users. Many statistical tools, custom metrics and sets for enrichment analysis.
Meta P-server [61]	http://metabolomics.helmholtz-muenchen.de/metap2/	Web-based	Compound IDs, sample meta-data	KEGG, HMDB, LipidMaps, PubChem	Data quality control, statistical analysis, hypothesis testing.	No use of organismal databases. Focus mainly on global statistical analysis.
MassTrix [62, 63]	http://metabolomics.helmholtz-muenchen.de/masstrix2/	Web-based	MS spectra	KEGG, HMDB, LipidMaps	Compound mapping	Choice of KEGG organism. Optional background set. Color-coding.
BioCyc (Pathway Tools) [52]	http://biocyc.org/	Installation required	Annotated genome, ‘omics data	MetaCyc	Network exploration, genome annotation, ‘omics data painting.	Comprehensive systems biology network analysis.
Pathos [64]	http://motif.gla.ac.uk/Pathos/index.html	Web-based	Simple m/z values, Compound IDs	KEGG	Compound mapping	Choice of limited organism databases.
PaintOmics [65]	http://www.paintomics.org	Web-based	KEGG formatted metabolites and/or genes	KEGG	Compound mapping	Choice of 100 hundred top species. Colours pathway metabolites and genes according to increase/decrease.
IMPaLA [66]	http://impala.molgen.mpg.de/	Web-based	Gene IDs and/or Compound IDs	KEGG, HMDB, CAS, ChEBI, PubChem, Reactome, Wikipathways	Enrichment Analysis	Combined analysis with proteins or transcripts. Organism independent. Optional background set.
MetaMapp [25]	http://uranus.fiehnlab.ucdavis.edu:8080/MetaMapp/homePage	Web-based	Compound IDs	KEGG	Metabolite networking	Organism independent. Network construction based on chemical similarity.
VANTED [67]	http://vanted.ipk-gatersleben.de/	Installation required	Compound abundances	KEGG	Metabolite networking, compound mapping, statistical analysis	Combined analysis with proteins and transcripts. Organism independent. Direct visualization of results on networks. Time course analysis. Statistical analysis.
TICL [68]	http://mips.helmholtz-muenchen.de/proj/cmp/home.html	Web-based	Compound IDs	KEGG	Enrichment analysis.	No choice of organism. Currently non-functional

Metabolomics secondary analysis tools have been developed by a number of groups with diverse implementations, however there are many commonalities. One of the major benefits of many of these tools is their implementations of user-friendly GUIs, allowing greater accessibility and precluding the necessity of learning the complicated tools they’re based on, most prevalently, R(The R Project for Statistical Computing, www.R-project.org). Before continuing, enrichment analysis will be used as a synonym for over-representation analysis, which some tools prefer to use. For enrichment analysis two objects are needed, a (ranked) list of items (i.e. genes or metabolites) provided by the experiment and a background set of annotations, derived from biochemical databases, computationally, through manual curation or some combination thereof. The list of metabolites can be ranked based on some metric indicating how different the abundances of each metabolite is between two sample classes, which can be calculated a number of different ways. As such the list will then show the metabolites with the most different abundances at the top of the list and the most similar at the bottom. The background set should contain all known metabolic pathways in an organism, each pathway including all the involved metabolites. For example the ‘TCA cycle’ contains the metabolites succinate, oxaloacetate, isocitrate etc. Compounds can occur multiple times as they are parts of many pathways, such as oxaloacetate which also appears in glyoxylate metabolism, among other pathways.

A danger with KEGG is that it includes pathways such as ‘metabolic pathways’ and ‘microbial metabolism in diverse environments’ which contain huge numbers of metabolites, and so as such are relatively meaningless when found as enrichment analysis hits. ‘Aminoacyl-tRNA biosynthesis’ is also a common hit to be taken with a grain of salt, as it is often highlighted when several amino acids are identified as significant. Careful scrutinization of metabolic pathways to ensure that they are logical is an important step in analyzing results produced by any platform. Other problems can arise when a dataset contains inordinate representation of certain pathways (either very few or very many). When many metabolites from one pathway are found in a dataset this pathway may be found to be significant mainly due to the large number of metabolites. Also the converse can happen if only a few metabolites are present in the dataset, but they changed significantly between classes, the pathway may not be found to be important due to the low number of metabolites [56]. Another issue occurs when querying the general (non-species specific) KEGG database as pathways that are non-existent in the experimental organism arise as significant. Sometimes this is obvious as with ‘synthesis of plant secondary metabolites’, though other times it may be difficult to know especially since well-curated metabolism databases exist for a scant few organisms. This is problematic for checking not only whether the pathway exists, but whether the annotation is accurate. Finally, even with well-annotated organisms there will metabolites identified that have not been assigned to any reaction whereas in poorly annotated organisms metabolites may be in metabolic pathways differently than expected from the canonical databases. Thus it is of the utmost importance for researchers to carefully regard the results produced by any secondary analysis tool and to understand how each piece of software works to ensure that the biological interpretation of the data is not skewed by some computational artifact. Cross-validating results through the use of multiple tools or multiple users producing the same result with a given platform is time consuming but would buttress the confidence in a result. Ultimately the best form of validation is a follow-up experiment however finding support from the literature for a result will also boost confidence.

*Metabolite mapping*: Visual attribution of specified metabolites within known, *pre-defined* metabolic pathways. Can include further information like significance and abundance in control vs. experimental classes.

*Metabolite Networking*: Statistical computation that groups metabolites together based on some property.

*Network Topology Analysis*: Statistical computation that computes how objects (nodes and edges) are related with a particular network graph.

*Enrichment analysis*: Statistical calculation that uses biological annotation to attempt to discern out of a set of (significantly changing) metabolites which higher level functional properties (pathways) are being affected.

These issues, and KEGG’s issues are somewhat alleviated by the BioCyc[45] series of organism specific databases, which if one has not already been generated, researchers with fully sequenced organisms can automatically produce such a customized database using the powerful Pathway Tools software [52]. This tool takes a sequenced, annotated genome and determines which metabolic reactions exist by comparing against the MetaCyc database of ‘all’ known metabolic reactions. A rudimentary metabolic network is then generated which must be manually curated using actual experimental knowledge to ensure that the computational model is actually accurate. These models have shown useful to many researchers, however their use is less prevalent among enrichment analysis tools.()

**Figure 1:**
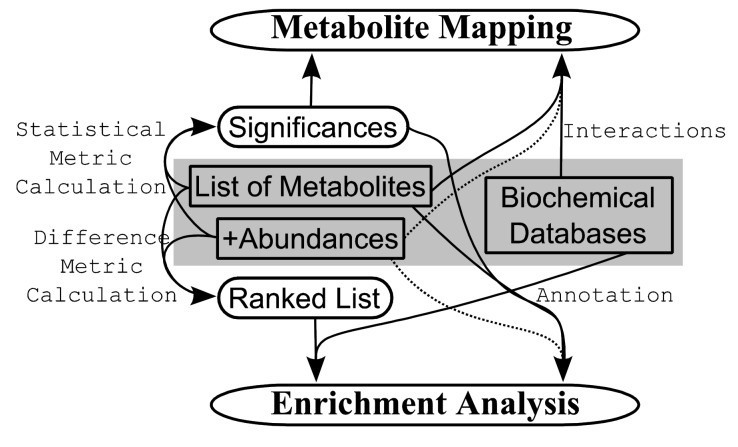
**Flow chart showing possibilities of metabolomics secondary analysis**. Beginning with a list of metabolites, and also in some cases associated with relative abundances or a comparable metric the data can be analyzed two different ways which may include intermediary steps. Also needed is a biochemical database to be used in annotating the biological (and/or chemical function) of the listed metabolites. The list of metabolites plus abundances can undergo statistical analysis in order to pre-screen for metabolites having significant differences between sample classes, or the abundances can be used to calculate how differently they’re expressed between each sample class (difference metric) which is then used to rank the list from most different to most similar. Significances can be used for both metabolite mapping and enrichment analysis. Metabolite mapping is the visual attribution of specified metabolites within known, *pre-defined* metabolic pathways which can include further information like significance and abundances as node attributes such as size and colour. Enrichment analysis is a statistical calculation that uses biological annotation to attempt to discern out the input metabolites which higher level functional properties (pathways) are being affected. This can take the form of searching for particular annotations at the top/bottom of the ranked list or examining whether a particular set is over-represented in the significant list.

## Overview of Metabolomics Secondary Analysis Tools

As with the rest of the metabolomics field, the sub-field of secondary analysis is rapidly evolving. Many tools for metabolite mapping and enrichment have been recently developed and are available for use. Generally these tools can be divided into two categories: enrichment analysis and metabolite mapping. Enrichment analysis aims to provide higher level information about metabolism from a list of metabolite abundances in different sample classes. Metabolite mapping provides a visual representation of metabolomic data by showing the identified metabolites (and their abundances) on a network graph, often obtained from a biochemical database. Some tools provide other functions as well, or can perform both simultaneously. Additionally the option to integrate other ‘omics data is becoming more prevalent. MetaboAnalyst and MeltDB are two platforms that warrant special mention as they provide a comprehensive environment to analyze metabolomics data from raw spectra all the way to secondary analysis. Finally it should be noted that the platforms discussed here are by no means an exhaustive list, merely a representative set of the most used and promising tools at this time.

### Comprehensive Platforms

#### MetaboAnalyst

MetaboAnalyst [57] provides a suite of utilities allowing comprehensive analysis from raw spectral data to pathway analysis within one platform. Also included are tutorials and example datasets that can easily be loaded to practice analysis. Five main choices are available: statistical, enrichment and pathway analysis as well as time course analysis. A number of other utilities including data quality checking (useful for batch effects) and a metabolite ID converter among others are also included. If beginning from raw GC or LC-MS data MetaboAnalyst uses XCMS [69] for peak fitting, identification etc. Once at the peak list (NMR or MS) stage, various preprocessing options such as data-filtering and missing value estimation can be used. Next a number of normalization, transformation and scaling operations can be performed. At this point the dataset is entered and can be subjected to MetaboAnalyst’s entire suite of statistical analyses including metabolomics standards like PCA, PLS-DA and hierarchically clustered heatmaps, among many other options. While all these tools are useful and highly convenient, they can similarly be performed by many other platforms, albeit often with less accessibility making MetaboAnalyst a good option for those new to the field. It is the secondary analysis tools MSEA and MetPa (accessible as enrichment and pathway analysis) however which are of interest to this review.

The Enrichment Analysis tool of MetaboAnalyst was one of the earliest implementations of GSEA for metabolomics datasets. As it stands, it is quite biased towards human metabolism as except for the custom option, all the available background sets for enrichment analysis are of various mammalian derived human-centric sets including blood, urine and disease associated metabolite sets. It is however possible to provide a custom background set thereby allowing any organism to be studied. This implementation of MSEA provides three options for input: a single column list of compounds (Over Representation Analysis, ORA), a two column list of compounds AND abundances (Single Sample Profiling, SSP) and a multi-column table of compound abundances in classed samples (Quantitative Enrichment Analysis, QEA). Each option can provide different information. ORA will calculate whether a particular set of metabolites is statistically significantly higher in the input list than a random list, which can be used to examine ranked or threshold cut-off lists. SSP is aimed at determining whether any metabolites are above the normal range for common human biofluids. QEA is the most canonical and will determine which metabolite sets are enriched within the provided class labels, while providing a correlation value and p-value. MetaboAnalyst’s MSEA has been used for a number of applications including aiding in characterizing the metabolic basis of Fragile X syndrome [70], understanding how various environmental pollutants affect goldfish tissues differently [71], and in identifying metabolic changes that occur as mice age [72]. Generally the results provided by MSEA were used to contextualize the observed changes in individual metabolites.

The Pathway Analysis tool of MetaboAnalyst, MetPa, performs somewhat similarly to MSEA, however it performs pathway enrichment and network topology analysis. It also provides broader options for organism databases including 17 common model organisms such as *C. elegans, A. thaliana, E. coli, M. musculus* among others, as well as a custom option. Input options are the same as MSEA. Once the data is loaded, the background database selected, the test method (Fisher’s Exact or Hypergeometric) and network topology metric must also be chosen. Output from MetPa is an interactive set of graphs. One graph plots the p-values vs pathway impact for the computed metabolic pathways. This graph allows one to discover the highest significantly impacted pathways for further exploration. Clicking data points on this graph will cause the network to be displayed in an adjacent view, with the input metabolites highlighted. Clicking these metabolites will show a box whiskers plot for each class allowing one to visualize increases and decreases. Through these functions MetPa can be used to visualize metabolomic data within known metabolic pathways, along with calculating which pathways are significantly affected. This ability makes it a powerful tool for secondary analysis. It has been used to understand the diabetes-contextual effects of a high-fructose diet in rats [73]. The results were used along with corroboration from TICL [68] to provide a starting point for analysis. MetPa has also been used to understand metabolomic results of renal injury in heart failure patients [74]. Finally it was used to identify perturbations to leucine and cysteine amino acid metabolism as well as energy metabolism in Dupuytren’s disease of fibroblasts from the palm of the hand [75]. This presence of a specific metabolic phenotype will aid in the pursuit of the cause of the disease.

#### MeltDB

MeltDB [30] is another comprehensive suite for metabolomics data analysis designed explicitly as a free and platform independent integrated project management and analytical pipeline that takes raw GC or LC-MS data through spectral preprocessing, data normalization, statistical analysis and recently integrated, enrichment analysis all within the same system. Their registration required web-based implementation allows multiple users (with various privileges such as view-only accounts) to work in parallel on the same projects. Also available are features for experiment meta-data description such as test conditions, extraction method and analytical parameters. MeltDB allows for data to be input at any level of processing, IE raw spectra can be imported and have their peaks detected, identified and quantified (with a choice from a variety of methods) or previously pre-processed data can be imported. This includes access to their implementation of MSEA [60]. Similar to MetaboAnalyst, MeltDB provides a wealth of options and utilities for analyzing MS metabolomics data including a large number of platforms (also accessible as stand-alone applications eg. XCMS [69]) for chromatographic MS processing. It is also able to import preprocessed data from common (non open-source) vendor-specific software. Further in the pipeline and similar to MetaboAnalyst, MeltDB can be used to perform statistical analysis like PCA and generate figures such as hierarchically clustered heatmaps which again is very useful for new comers. These features have been used in demonstrating how SEC is a superior sample collection method for Corynebacterium glutanicum and in the metabolic characterization of different parts of the grain during the highly important process of industrial barley malting [76]. MSEA takes a ranked list of compounds and determines whether a particular pathway is enriched towards the top or bottom of this list, however it provides the highly-convenient option of being able to natively rank the list based on a number of metrics. In their analysis used to test this new tool it was found that the use of a highly specific background set, CglCyc (from their sequenced *C. glutanicum*), which was automatically produced then manually curated produced better results than the use of the KEGG database. Two main reasons were provided. First, KEGG pathways are much larger and interconnected than their CglCyc pathways resulting in the obstruction of information when there are opposite fluxes in different parts of the pathway (the provided occurrence was in opposite abundances in the upper and lower regions of gluconeogenesis). Second, as previously noted, KEGG annotated pathways do not exist in all organisms. Further testing was performed on datasets obtained from a number of mutant lysine production strains, which generally showed the expected results of alterations to lysine and threonine metabolic pathways as their mutations targeted this split of branched chain amino acid metabolism. This new tool appears to be quite powerful, though also very reliant on the quality of the background set.

### Enrichment Analysis

#### PAPi

Pathway Activity Profiling [24] is an R-based tool designed specifically for secondary analysis of metabolomic data. As input it takes a list with abundances (normalized and scaled) and working on the assumptions that the detection (IE presence in the list) of more metabolites in a pathway and that lower abundances of those metabolites indicates higher flux and therefore higher pathway activity PAPi calculates an activity score (AS) for each pathway. The metabolic pathways are taken from the general KEGG database and the AS indicates the probability of this pathway being active in the cell. These scores can then be used to compare experimental and control conditions by performing ANOVA or a t-test to compare two sample types. As such, PAPi is a classic implementation of metabolomics secondary analysis, allowing users to derive higher level information from a simple list of metabolites. It has been used to show the similarity between genetic and environmental perturbation of yeast strains, which was in agreement with the previously published conclusions. It has also been used to show that sound caused frequency dependent metabolic alterations^77^ and that different biological interpretations will be made in microbial metabolomics based on the extraction methodology [78]. While PAPi’s assumptions may not be universally accurate (TCA cycle intermediates can have high abundance even when flux through the reactions in this pathway is also high) and the interface is more difficult than other platforms, it still provides an excellent option for enrichment analysis.

#### MBRole

Metabolic Biological Role [27] is another classic implementation of enrichment analysis. Taking as input a list of significantly changing metabolites (IE statistically processed already) MBRole calculates which pathways and chemical groups are enriched either against a pre-compiled (from KEGG) or user supplied background set. Output is a table of metabolic pathways with significance p-values and the pathways hyper-linked to KEGG metabolism maps. MBRole is an easy to use yet powerful tool as it can take input under many different database formats and compute the enrichment based on any of the available annotations. Also the use of any of the organism-specific KEGG annotations makes the investigation of diverse organisms easy. It has been used as a starting point to interpret steatotic liver tissue metabolomic data. Results were interpreted in the context of the identified enriched pathways that were altered in steatotic tissue with prior knowledge and direct examination of the metabolite pools[79]. While the flaws of KEGG annotations remain present, MBRole provides an excellent simple implementation of enrichment analysis for the average user.

#### MPEA

Metabolite Pathway Enrichment Analysis [29] is a stand-alone tool that takes a ranked list of metabolites (either KEGG IDs or mass spectra with retention index) and determines if a particular known metabolic pathway (as annotated in the background set) tends to appear more towards the top or bottom of that list. Output is a table of metabolic pathways (linked to KEGG) with p-values (among other data) indicating whether the pathway was significantly enriched. The default settings are human-biased as KEGG and the SMPDB[80] (a curated set of human pathways) are queried however a custom background set option is also available. The list can be ranked by any metric, such as significance to a model or t-tests of concentration. One of the main differences between MPEA and other tools is the ability to work with ambiguously identified compounds, especially useful when working from mass spectra. Mass spectra are first identified using the GMD, then ambiguous identifications resolved within the pathway enrichment analysis. This tool has been used by the group that developed it to make a minor contribution in studying Alzheimer’s progression, showing that pentose-phosphate pathway was altered in patients that were developing dementia [81].

#### TICL

The Tool for automatic Interpretation of a Compound List [68] is an early example of metabolomics secondary analysis. It was designed to take a list of (significantly) changed metabolites from an experiment and calculate whether they are biologically related, according to KEGG pathways. Taking a list of KEGG IDs as input, TICL outputs a list of pathways with p-values indicating the probability of this pathway appearing by chance. A relatively underused tool, TICL has been used to demonstrate differences between the biofilm and planktonic response to metal stress[82], and to supplement/compare MetPa results in studying the effects of a high-fructose diet on rats[73]. An early pioneer in the field with a sound premise, at the time of writing TICL was not functional.

#### IMPaLA

Integrated Molecular Pathway-Level Analysis [66] is a tool designed to perform enrichment analysis on both metabolomic and proteomic or transcriptomic datasets simultaneously. Taking as input a list of metabolites plus a list of genes/proteins if available (not necessary) IMPaLA can calculate pathway enrichment using one of two methods. Enrichment is computed either against a user-provided background set or against the whole set chosen from the available input format databases (KEGG, HMDB, ChEBI etc). The input can either be preselected for significance by some other analysis or can include abundance information between two different classes. In either case, output is a table of pathways hyper-linked to the database it was found in along with a p-value indicating significance. For the purposes of combined analyses there is a p-value calculated separately for genes and metabolites as well as a combined value. This makes IMPaLA a good tool for analyzing combined datasets however the limited outlinking with pathways and lack of visualization means that there are potentially better options for just metabolomics enrichment analysis.

### Metabolite Mapping

#### MetaMapp

MetaMapp [25] presents a novel approach to metabolic mapping which uses chemical similarity of compounds in order to overcome the difficulties of missing, unknown and unannotated metabolites prevalent in metabolomics data. Development of this platform was due to a dissatisfaction with other available metabolic mapping tools, generally due to the above being more or less addressed, depending on the particular tool. Hence MetaMapp was developed on the premise that since biochemistry is the interconversion of chemically similar entities, compounds can be clustered solely by their chemical similarity. While this was found to be highly beneficial for metabolites without reaction annotation, chemical similarity mis-clustered some obviously biologically-related metabolites. As such MetaMapp uses both chemical similarity and KEGG reactant pair data. Finally, the problem of unknown compounds was addressed by adding the possibility to map metabolites based on their mass spectral similarity. While the resultant graphs are somewhat busy, especially when statistical information such as significance or fold-change is applied to node attributes such as size and colour, this novel approach can provide much needed contextual information about unannotated and unknown metabolites. The function of this tool was demonstrated using GC-MS metabolomic data from three tissues involved in fetal exposure to tobacco smoke: maternal plasma and lungs and fetal lungs. Using MetaMapp, an identical network of the 179 identified metabolites (excluding unknowns) was generated for each tissue, with various biologically and chemically related clusters clearly visible. For each graph, only significant metabolites were labeled, with color representing up or down regulation (compared to the unexposed control) and size representing fold-change. Aligning these three graphs allowed for a visual inspection of the metabolomic data which made interpretation pleasantly obvious. These results clearly showed that the fetal lungs were most affected, with fatty acids being the most dysregulated. Also present were alterations to several amino acids. These results show the promise of this novel technique in interpreting metabolomics experiments. One of the most exciting features, which was not involved in the confirmatory results is the ability to map unknown metabolites. This possibility will likely be very useful in discovering novel metabolic pathways in the future.

#### MassTrix

MassTrix [62] is a platform for automatically identifying high precision spectra and mapping data in the context of organism-specific KEGG pathways. It is one of the oldest tools discussed and has been well-utilized. Developed by the same group as Meta P-server, the ability to integrate raw transcriptomic data was recently added [63]. This ability, plus the identification of compounds previously annotated within an organism (from KEGG) differentiates MassTrix from other platforms. The identification procedure is based on comparing the masses of input ions to known metabolites obtained from their multi-integrated database including options for adducts and isotopes though it may be by-passed by entering previously identified KEGG IDs. Once data has been uploaded and analyzed, two sets of results are provided. The Compound section shows all of the annotated compounds with mass, formula, identity and which database the ID was acquired from. This section can be examined for ambiguity issues and compounds are clickable to find their pathway annotations linked from KEGG. The Pathway section of results allows pathways of interest (those which include identified compounds) to be visualized with ID’d compounds highlighted as well as transcriptomic data applied. One drawback here is the inability to assign metabolite abundances. Indeed, MassTrix is somewhat limited compared to more modern tools, though the added integration of transcriptomic data has great potential. Additionally, MassTrix has an excellent track record of use for a wide variety of applications. It has been used to study the effects of dry-bean consumption on carcinogenesis in rats^83^, to explore the wide dynamic range of the human metabolome in healthy individuals^84^ and also quite interestingly to study the ‘metabolome’ of organic matter in sea-spray [85] among many other successful applications

#### PaintOmics

PaintOmics [65] is an ‘omics mapping web-tool that takes metabolite and transcript abundances and significances and maps them onto organism-specific KEGG maps. Taking either or both types of data, PaintOmics will produce a series of KEGG pathway maps with the data highlighted on the networks, as well as providing an enrichment analysis p-value for each pathway. It is capable of coloring objects (metabolites or transcripts) for each condition provided in the input. Given that PaintOmics will include any pathway with at least one entry the enrichment analysis or prior knowledge will be needed to assist in interpretation. While an improvement over the combined mapping abilities of MassTrix, PaintOmics suffers from the same drawbacks of other KEGG based utilities, namely quality of annotation and size of pathways, both of which are addressed by MetaMapp. Still the ability to seamlessly integrate both transcriptomic and metabolomic datasets, as well as display the results of multiple classes in one visualization make it a useful tool. Additionally as KEGG annotations continue to improve and include more species it will only increase in utility. Thusfar it has be used to map transcriptomic data from differently cultured hepatocellular carcinoma cells [86].

#### VANTED

The tool for the Visualization and Analysis of Networks with related Experimental Data (VANTED) [67] is another tool capable of mapping ‘omics data onto custom and KEGG derived networks with additional visualization and analysis options. Contrary to most other programs, VANTED must be installed on the user’s computer. Taking any combination of data (in the form of relative or absolute concentrations in different samples), it will present the data upon the relevant biological networks. This allows users to see the concentrations of metabolites in sample classes AND their connection to other metabolites and/or genes and/or proteins together. This works best when many linked metabolites have been quantified, which is unfortunately often not the case in metabolomics experiments. Statistical tests indicating whether metabolite concentrations are significantly different from the control can also be automatically performed and their result appended to the visualization. VANTED provides numerous options for how the networks are generated including downloaded organism-specific KEGG maps as well as correlation-based mapping using various metrics. These statistically oriented maps, along with the convenient presentation of metabolite abundance data make VANTED a powerful tool for metabolomic secondary analysis. Since its release it has been updated frequently and has been highly used in the field. VANTED has been used to interpret metabolomics results in a wide variety of studies including the effects of drought response on wheat leaves [87], the effects of pyruvate for treatment of mitochondrial disease [88] and understanding how glucose starvation affects *Staphylococcus aureus* [89], among many others.

#### Pathos

Pathos [64] is a metabolite mapping tool designed in response to MassTrix’s limitations. Specifically it was made to include the ability to map data from different experimental conditions and compare their degree of change. Apart from this difference, Pathos identification functions similarly to MassTrix taking mass/charge values (or previously identified compound IDs) and identifies them using an organism-specific KEGG database, then displays the KEGG pathways with the input metabolites highlighted. Different than MassTrix though, a p-value for each pathway is not provided. Output is a list of pathways with the number (out of the total) of identified metabolites which are clickable to show the mapped pathway. On the visualization identified metabolites can be clicked to show a column plot comparing the abundances under each condition. Generally this tool is relatively comparable to the many other metabolite mapping tools. It has been used in conjunction with Ingenuity Pathway Analysis to monitor stem cells in regenerative medicine [90].

#### ProMeTra

ProMeTra [91] is an ‘omics viewing web-tool designed to visualize any kind of ‘omics data not only on KEGG database derived metabolic pathways but also on user supplied pathways. Its visualization system was designed to take advantage of the Scalable Vector Graphics (SVG) format allowing easy coloring (eg by abundance differences), extra annotation and even the production of animations. These features allow for the easy generation of clear, visually appealing multi-class annotated pathway maps for use in biological interpretation. Regulons can also be visualized, which when annotated with transcriptomic can clearly show biological effects. ProMeTra’s main draw compared to other mapping tools is the use of SVG graphics which allow for infinite zooming, output at any resolution and easy manipulation in SVG capable drawing programs. Even so it is an underused tool, perhaps due to the login-based (but not required) system or the less intuitive UI.

### Others

#### MetExplore

MetExplore [28] is a metabolism exploration suite which can analyze metabolic networks without metabolomic data, though it also has a tool which will identify all the pathways individual metabolites can be involved in. This implementation was designed to overcome MetaCyc’s shortcomings of mapping compounds iteratively onto each relevant pathway, instead MetExplore aims for one single representation of each metabolite. Using the MetaCyc/BioCyc series of databases there is a relatively wide choice of organism databases. MetExplore’s main tools are Metabolome Mapping and a series of computational analysis tools. These tools do not involve data input, they just provide a variety of methods to analyze MetaCyc derived metabolic networks. Choke point analysis can identify reactions/metabolites that are unique within the network whereas scope and precursor analysis allow the investigation of what metabolites are required/are possible to produce the other metabolites in the network. Such analyses can be used to identify a minimal set of media or whether a particular metabolite can be generated given a defined media. These tools have been used to work on understanding the symbiotic relationship between *Buchnera* and its aphid host [92]. For all types of analysis filters are available to restrict artifacts and adjust the analysis. MetExplore’s metabolome mapping tool is somewhat more limited than other comparably named tools. It can take as input a list of masses or identified metabolites, but does not output a visualization. Instead it provides a table view which indicates for each metabolites which metabolic pathways they are involved in and also topological information IE the number of reactions that produce/consume it, ranging from none to many for each direction. While this has its uses, the same information can generally be obtained from other mapping tools, however the computational analysis tools provided by MetExplore could be quite useful to researchers working on organisms that have a –Cyc database.

#### Meta P-server

Meta P-server [61] is a metabolomics exploration tool specifically designed to work with multi-class experiments. Taking as input a metabolite quantitation table and a sample description matrix, a number of statistical tests are automatically performed which can then be viewed and colored according to any identifiers in the sample description matrix. This allows the quick and easy checking for batch effects, outlying samples and also overall data quality. The two main statistical outputs are PCA plots and hypothesis testing. The generated PCA plots can be colored by each possible class identifier allowing the most important classifier to be quickly found. Hypothesis testing of whether metabolite concentrations are different is performed for each possible class division, generating a series of boxplots with significant differences highlighted. For example if the data are classified by sampling day (Day 2, 3 and 5) and by drug dosage (none, high) two sets of hypothesis test results will be produced, one showing whether there are differences between concentration between each day for a given dosage, and the other showing differences between each dosage for a given day. This is performed for each metabolite. The other statistical result generated is a heatmap of correlations for any given numerical classifiers. Also included an option for direct import of Biocrates AbsoluteIDQ [5] kit derived data. While not providing any secondary analysis options, Meta P-server provides a quick and easy method for statistical analysis of multi-class experiments.

### Commercial Software

All of the above software are completely free to use, with most not even requiring registration of an account. There are also a number of commercial pieces of software available. These software are generally designed to be comprehensive solutions for use in multi-omics experiments including a number of integrated pathway analysis and contextual visualization choices. Additionally these software are more geared towards human/mammalian model (i.e. mouse, rat) disease and drug investigations and use manually curated proprietary databases.

#### IPA

Ingenuity Systems Inc. (Redwood, CA), offers a data analysis suite deemed Ingenuity Pathway Analysis (www.ingenuity.com). Using their Ingenuity Knowledge Base, metabolomic data (among other ‘omics data types) can be mapped onto networks and enrichment analysis can also be performed. These features are among a large suite of systems biology analysis tools that are designed to allow biologically contextual representation of data. These data can come from a large variety of different types of experiments ranging from small-scale drug target experiments to combined transcriptomic and metabolomic studies. While many types of data can be used, IPA can take just a list of altered metabolites as input and use literature-characterized signaling and metabolic pathways to identify the biologically relevant effects of an experiment. This approach has been used to aid in the understanding of diverse metabolomics experiments, including colorectal cancer [93] and detoxifying processes in traditional Chinese medicine [94].

#### MetaCore

GeneGo Inc. (Carlsbad, CA), of Thompson Reuters, along with MetaMiner and MetaDrug is a series of pathway analysis and data mining tools highly geared towards human disease investigations. Among these tools are features for mapping multi-omics experiments, drug target prediction, and pathway perturbation analysis for toxicity studies. These tools all work against their proprietary manually curated databases, which for some features extend into common model and pathology-relevant organisms. Metacore has been used to perform over representation and network analysis on datasets combining metabolomic and either transcriptomic or proteomic data to understand toxin mode of actions [95] and biomarkers in colorectal cancer [96], respectively.

#### GeneSpring

Agilent Technologies (Santa Clara, CA) provides a comparable suite while also providing features for MS analysis of raw data by integrating with their Mass Profiler Professional, however this program has mostly been used for transcriptomic analysis.

## Summary and Outlook

Metabolomics represents the apical step in the paradigm of systems biology. Its rapid development has provided unparalleled understanding of metabolic processes to a plethora of different fields. The secondary analysis of metabolomics data is a recent addition which will provide researchers with much more power in finding biological interpretation. Currently though, few researchers tend to be using enrichment tools and the provided results are rarely heavily discussed in their manuscripts. While the results from this type of analysis should not be the sole source of information for biological interpretation of metabolomics experiment, the provided results are highly useful in giving metabolic context for many metabolites at once, without having to search through databases one at a time. Still, a number of researchers have shown they provide an excellent springboard for diving into the depths of metabolomics data.

As the field of metabolomics secondary analysis evolves, a number of challenges remain. Beyond the ever-changing processes of data pre-processing and statistical analysis for metabolite significance, the contextual interpretation of metabolomics results will also need improving. While the software described in this review are a good beginning, future analyses will need to be highly tailored towards organism-specific metabolic reconstructions. The MetaCyc derived series of databases have begun to fill this role, however as genome annotation remains a marginally accurate process, the models generated from such data are affected equally. Community driven confirmation and elucidation of genetic and metabolic annotation of databases like EcoCyc have shown that it is only a matter of time and effort for reasonable computational models to be built. For other organisms comparable tools will be developed as the research community deems them important. Another major improvement to be made would be decreasing the number of inaccurate and meaningless pathway hits made in enrichment analysis. This may be difficult though as metabolites will only become annotated into more metabolic pathways as their connections are elucidated. Great steps have been made in metabolite mapping techniques, especially with the chemical similarity connections provided by MetaMapp, however the interpretability of all maps remains difficult. Computational improvements in the graphical presentation and ease of producing legible maps will make metabolite mapping better for metabolomic secondary analysis.

Achieving good biological interpretations of metabolomics data is easier in medical studies of humans and generally when using highly studied model organisms due to the preponderance and quality of databases associated with these subjects. While it is generally true in science that studying organisms with a wealth of literature makes data interpretation easier, this effect is greatly amplified in metabolomics. Thus humans, *E. coli*, and *A. thaliana* are much more convenient to perform metabolomics secondary analysis upon compared to, for example, a freshly isolated environmental bacterial strain. Again MetaCyc and its associated tools present the solution though producing an organism-specific –Cyc database from an annotated genome remains a complex time consuming endeavour. Fortunately the documentation continues to improve and SRI International is well-engaged with the research community for educational and software feedback purposes.

Regarding the current set of available tools, they all have strengths and weaknesses and it should not come to the use of one over the other. Once a metabolomics dataset has reached the point of secondary analysis, applying any of the above tools is not a hugely time-consuming process and so it may be wise to use multiple tools and take the consensus results. Definitely the use of at least one enrichment analysis and one visualization/mapping tool is recommended. Given the complexity of metabolomic data, it is also important to carefully regard the results from secondary analysis as it is possible for enrichment analysis to produce significant pathway hits from only one or two metabolites in a pathway. As such, careful scrutinization and logical biological interpretation of the data must be undertaken. With this in mind metabolomics researchers should strive to integrate secondary analysis into their studies as these highly useful results can be obtained very rapidly. Clearly the field of secondary analysis is coming into its own and its continuing development will only serve to improve the success of the metabolomics approach.
